# Silk from Crickets: A New Twist on Spinning

**DOI:** 10.1371/journal.pone.0030408

**Published:** 2012-02-15

**Authors:** Andrew A. Walker, Sarah Weisman, Jeffrey S. Church, David J. Merritt, Stephen T. Mudie, Tara D. Sutherland

**Affiliations:** 1 Evolution, Ecology and Genetics, Research School of Biology, Australian National University, Canberra, Australia; 2 Ecosystem Sciences, Commonwealth Scientific and Industrial Research Organisation, Acton, Australia; 3 Materials Science and Engineering, Commonwealth Scientific and Industrial Research Organisation, Belmont, Australia; 4 School of Biological Sciences, University of Queensland, Brisbane, Australia; 5 Australian Synchrotron, Clayton, Australia; Massachusetts Institute of Technology, United States of America

## Abstract

Raspy crickets (Orthoptera: Gryllacrididae) are unique among the orthopterans in producing silk, which is used to build shelters. This work studied the material composition and the fabrication of cricket silk for the first time. We examined silk-webs produced in captivity, which comprised cylindrical fibers and flat films. Spectra obtained from micro-Raman experiments indicated that the silk is composed of protein, primarily in a beta-sheet conformation, and that fibers and films are almost identical in terms of amino acid composition and secondary structure. The primary sequences of four silk proteins were identified through a mass spectrometry/cDNA library approach. The most abundant silk protein was large in size (300 and 220 kDa variants), rich in alanine, glycine and serine, and contained repetitive sequence motifs; these are features which are shared with several known beta-sheet forming silk proteins. Convergent evolution at the molecular level contrasts with development by crickets of a novel mechanism for silk fabrication. After secretion of cricket silk proteins by the labial glands they are fabricated into mature silk by the labium-hypopharynx, which is modified to allow the controlled formation of either fibers or films. Protein folding into beta-sheet structure during silk fabrication is not driven by shear forces, as is reported for other silks.

## Introduction

The ability to produce silk has evolved in at least 23 groups of insects [1], in spiders [2] and in several other arthropods [3], [4]. Silk research has focused on silkworm cocoon and spider dragline silks, which have independently evolved a number of convergent features. Spider and silkworm silks consist of long, repetitive proteins that fold predominantly into beta-sheets, with the protein backbone parallel to the fiber axis [2]. Highly ordered nanocrystals are embedded in regions of less order and confer high tensile strength to the fibers [5]. The molecular arrangement in spider and silkworm silks is the result of shear forces and controlled dehydration acting on highly concentrated silk protein solutions as they pass through a hardened aperture known as a spinneret [6], [7]. Although less characterised, other silks are dramatically different. For example, protein backbones in silks made by glow-worms and adult lacewings are orientated perpendicular instead of parallel to the fiber axis [8]; the silks of fleas, bees and lacewing larvae contain proteins arranged in alpha-helices instead of beta-sheets [8], [9]; and the fibrous proteins in some silks are an order of magnitude smaller than spider dragline and silkworm cocoon silk proteins [10]. Further characterisation of silks in addition to spider and silkworm silks will allow a comparative approach to understanding the complex molecular arrangements found in silk.

Crickets in the family Gryllacrididae (raspy crickets) produce silk, while only one other insect in the order Orthoptera does so [11], [12]. Raspy crickets use silk fibers to build shelters into which they retreat during the day [12], [13]. The fibers are used variously to sew leaves together, to stabilise burrows in earth or sand, or to restrict access to tree hollows depending on species [12], [14]. The shelters are generally presumed to be a defense against predation, though it has also been suggested that they may limit desiccation in drier environments [12]. Both sexes are capable of producing fibers within hours of hatching and continue to produce shelters throughout their lives [15]. Shelters are highly valued and individuals may label their own shelters with a chemical cue [16] allowing them to return to the same shelter many times.

Very little is known about the method of fabrication of silk fibers by raspy crickets. Rentz and John [12] observed silk production from cricket mouthparts, but the origin of the material is unknown and the internal anatomy of raspy crickets is poorly described. Other insects that generate silk from their mouthparts do so using protein solutions produced in modified labial glands [17]. Wetas and king crickets (Anostostomatidae), the closest relatives of raspy crickets [18], use their labial glands to produce saliva [19]. Anostostomatid labial glands are arranged in grape-like clusters called acini [20]. Acinar cells secrete into the lumen of a branching series of ductules joined to the common duct on each side of the body. The left and right common ducts join at the labium, where they empty into a cavity between the labium and hypopharynx, called the salivarium. An additional organ, the reservoir, is formed by a sack-like outgrowth of the common duct on each side [19], [20].

Nothing is known about the material composition of raspy cricket silk fibers or how they are produced. We investigated the biochemistry and physical structure of raspy cricket fibers and the method of their production. Our motivation in this work was to enhance understanding of which features of different silks that have evolved independently in different arthropod groups are convergent and functional, and which features are historical and accidental.

## Results

### Raspy cricket build shelters by using silk fibers and films to join other materials

**Figure 1:**
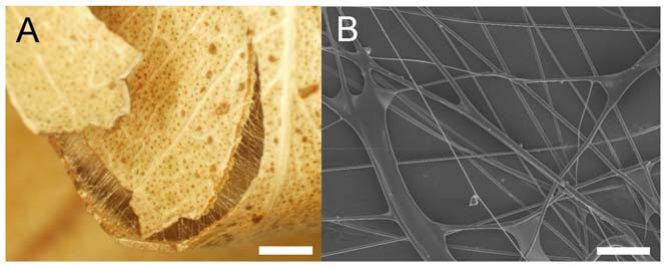
Raspy cricket silk-webs. (*a*) Shelter of silk and dry leaves made by *Hyalogryllacris*. Scale bar is 2 mm. (*b*) Scanning electron micrograph of *Apotrechus* silk-web showing cylindrical fibers and films. Scale bar is 100 µm.

Captive crickets built shelters by binding leaves or plastic card together with webs of silk (figure 1a). The fibers were cylindrical and uniform, with the diameter increasing as the crickets increased in size. For example, the diameter of fibers produced by a 16 mm long early instar *Apotrechus* were 4.2±1.1 µm. Seven months later, the same animal, measuring 48 mm, was producing fibers with diameter 12.4±2.1 µm.

Shelter construction behaviour was similar regardless of whether natural or artificial building materials were supplied. The process is shown in video S1. Silk production began by the cricket touching its labium to the surface of a leaf or piece of plastic, and depositing a film of silk material. As the labium was drawn away from the film, a fiber was produced, which was attached to another piece of building material with another film. Repeating this process resulted in a network of fibers joining the two pieces of building material. As successive layers of fibers were added, films were produced not only to secure fibers to building materials but also where fibers crossed, gluing them together (figure 1b). The end result of the building process was a ‘silk-web’ that served to seal an entry point to the cavity and to hold the building materials together. Most crickets constructed a shelter within 24 hours of being housed, incorporating additional fibers over successive days. To exit the shelter to forage, an access hole was cut through a silk-web using the mandibles. Access holes were sealed with a fresh silk-web after the insect returned. The insect ceased to produce fibers if the shelter was undisturbed for long periods, whilst removal of a shelter resulted in the construction of a replacement.

### Fibers and films have a similar molecular structure

**Figure 2:**
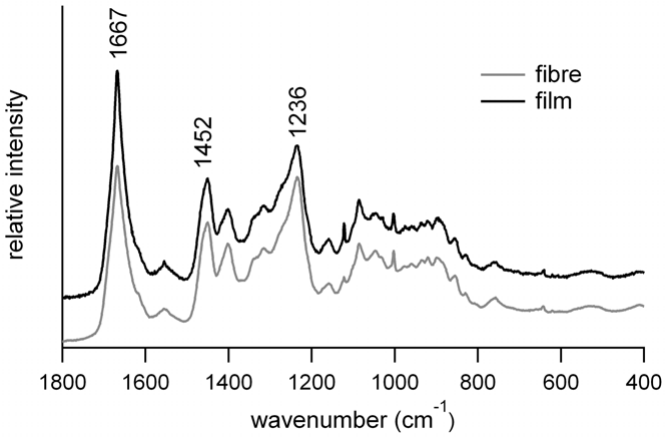
Raman spectra of raspy cricket fibers and films. Amide peak positions are indicated.

We investigated the chemistry of *Apotrechus* silk using Raman spectroscopy. Spectra obtained from fibers and films indicated a highly proteinaceous material, with no peaks attributed to chitin or other substances (figure 2). Spectra from films were essentially the same as those obtained from fibers indicating that the two materials contained proteins with the same secondary structures. Strong peak vibrations at 1667 cm^−1^ and 1235 cm^−1^ indicated that the dominant protein conformation present was beta-sheet [21] and the weak shoulder at 1259 cm^−1^ was due to disordered protein. Raman spectroscopy is sensitive to a number of specific amino acids and their environments within the protein structure, particularly tyrosine, tryptophan, phenylalanine, histidine, proline, hydroxyproline, methionine and cysteine [22], [23]. No significant difference in the relative strengths of frequencies attributed to any specific amino acid were detected in the spectra obtained from fibers and films, suggesting that the fiber and films had the same amino acid composition.

Fibers were anisotropic, having a birefringence of 0.013±0.002 in the long-slow direction, suggesting that protein chains in fibers are aligned relative to the axis of the fiber. Birefringence was not detected in films, but because films are much thinner than fibers it is unclear if this is because films are isotropic or because our instruments were not sensitive enough to detect their birefringence. Raman spectra obtained from various regions of the film randomly orientated with respect to any adjoining fibers were found to be identical. As the laser is polarized, this infers that there is no protein chain alignment in the films and that films are anisotropic at the molecular level. Silk proteins in beta-sheet conformation can be aligned with the peptide backbone either parallel (extended beta-sheet structure) or perpendicular (cross beta-sheet structure) to the axis of the fiber [8]. The measured WAXS pattern of cricket silk with the fiber axis vertical showed the strong arc attributed to the spacing between beta-strands in the direction of hydrogen bonds on the equator (table 1 and figure S1). This indicates that crystallites in cricket silk are arranged in an extended beta-sheet structure. The placing of another equatorial arc, attributed to the (2 2 0) reflection, depends in part on the inter-sheet spacing between amino acid side groups. Calculations from the position of this reflection suggests that in *Apotrechus* silk the inter-sheet spacing is 1.27 nm.

**Table 1:** Assignment of WAXS peaks.

peak position	d (nm)	assignment	physical correlate	expected d (nm)
equatorial	0.461	2 0 0	inter-strand spacing in direction of H-bonds	0.472
near-equatorial	0.373	2 0 1	inter-strand spacing in direction of H-bonds; amino acid spacing in direction of peptide backbone	0.383
equatorial	0.373	2 2 0	inter-strand spacing in direction of H-bonds; inter-sheet spacing in direction of side-chains	0.373^1^

1 The expected d-spacing for the (2 2 0) peak is 0.373 nm when the inter-sheet spacing in the direction of amino acid side-chains is 1.27 nm.

### Silk is produced from acinar labial glands

**Figure 3:**
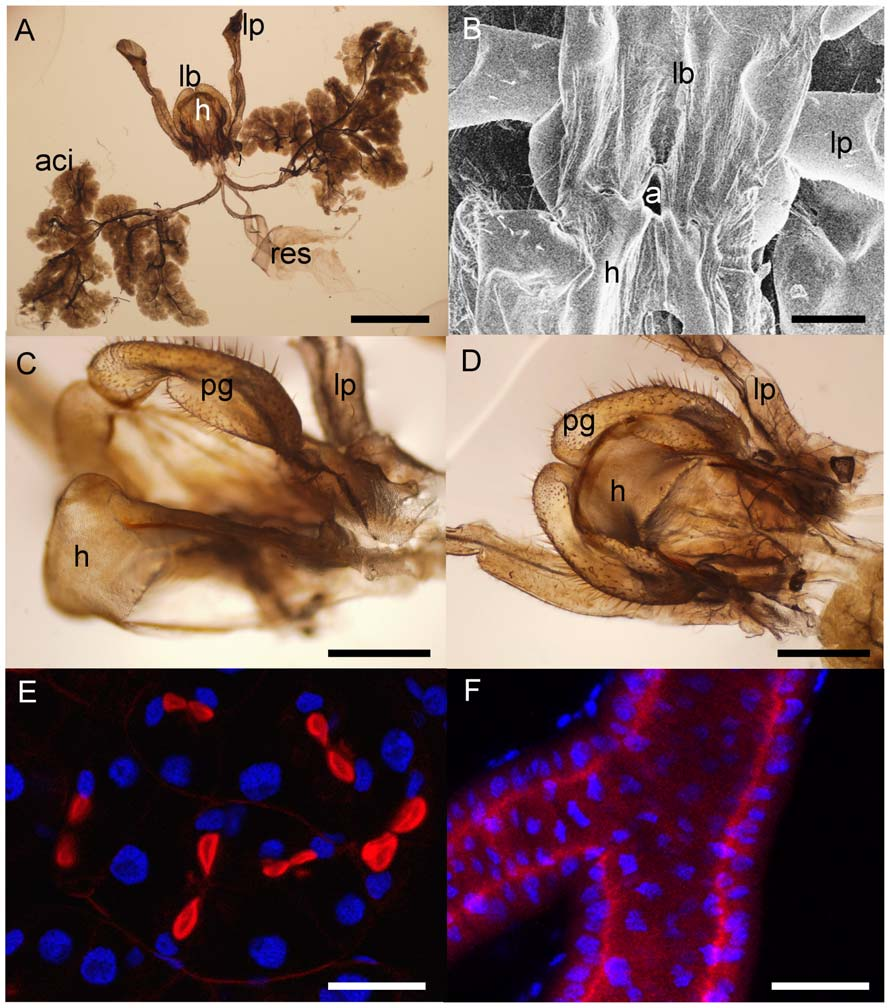
Raspy cricket labial glands. (*a*) Labial glands and labium of *Hyalogryllacris*; scale bar is 2 mm. (*b*) Scanning electron micrograph of a preparation from *Apotrechus* in which the hypopharynx has been pinned back to show the aperture through which the labial glands empty into the salivarium; scale bar is 500 µm. (*c*) Labium and hypopharynx in open position, showing the raised margins on the labial paraglossae of *Hyalogryllacris*; scale bar is 500 µm. (*d*) Same preparation as (*c*), labium and hypopharynx in closed position, with raised margins of paraglossae overlapping hypopharynx; scale bar is 500 µm. (*e*) Confocal slice through labial acinus of *Apotrechus* showing paired arrangement of nuclei (blue) and secretory invaginations (red); scale bar is 50 µm. (*f*) Projection of 18 images in z-series showing small cuticle-secreting cells lining the acinar labial duct of *Apotrechus*; scale bar is 50 µm. aci = acini; res = reservoirs; lb = labium; h = hypopharynx; lp = labial palp; a = aperture of labial glands into salivarium; pg = paraglossae of labium.

Silk made out of protein must have a glandular origin. Visual observations during dissection of *Apotrechus* and *Hyalogryllacris* revealed large, acinar labial glands in the thorax and head in a similar arrangement to those of anostostomatids (figure 3a). Ductules from the acini on each side of the body merged into a common duct ending at the labium. Whereas the reservoirs of anostostomatids are connected to the common acinar duct at some distance from the labium [20], raspy cricket reservoirs are joined to the end of the common duct within the labium. At this point the reservoirs and the common ducts from both sides of the body join together and empty into the salivarium through a common aperture. The size of this aperture was much larger than the diameter of silk fibers, measuring in excess of 100 µm across in adult *Apotrechus* (figure 3b). No structure similar to the hardened, external spinneret of lepidopterans was observed. Instead, the labium was similar to crickets that do not produce silk, with hypopharynx, glossae and paraglossae [24], except that the paraglossae had an unusual shape: the margins of the paraglossae were raised up into ridges that overlap the edges of the hypopharynx, so that the hypopharynx fits ‘hand in glove’ into the labium (figure 3c and 3d). To compare the structure of the labium of crickets that do not produce silk, we dissected a field cricket (*Acheta domestica*), a katydid (*Conocephalus* sp.) and an anostostomatid (*Penalva flavocalceata*). In these species, the surface of the paraglossae is flat or concave, without the distinctive raised margins that occur in raspy crickets.

Secretory cells have a distinctive morphology, with actin-rich secretory membranes and large or multiple nuclei [25]. To investigate the function of the acini and the reservoirs, we stained them with fluorescent dyes that reveal nuclei and the actin cytoskeleton. Acinar cells were organised in pairs, possessed a single large nucleus, to which an actin-rich invagination was closely apposed, consistent with a secretory role (figure 3e). The lobular, actin-rich lumen of each cell conjoined to a common lumen with a stellate, actin-rich periphery that was continuous with the common duct. Each acinus was composed of approximately 20 of these conjoined cells. The ducts were lined with smaller, flattened, cuticle-secreting cells, the luminal surface of which was actin-rich (figure 3f). The reservoirs consisted of a single layer of the same small cuticle-secreting epidermal cells, suggesting a role as a storage organ.

The proteins present in fluid-filled reservoirs were investigated by comparing the sequences of peptides following tryptic digestion to GenBank's non-redundant protein database and *in silico* translated sequences from our own *Apotrechus* cDNA library using LC/MS. Apart from house-keeping proteins, the only protein identified was amylase, an enzyme that breaks down the plant polymer starch (table S1). This suggests that reservoirs store saliva. As none of the raspy cricket silk proteins (described below) were detected in the reservoirs, it is unlikely that they store silk proteins.

### Raspy cricket silk proteins

The raspy cricket silk proteins were identified using a cDNA library/mass spectroscopy approach that has been applied to other silk-producing species [26]. We constructed a cDNA library of 3.5×10^5^ clones with an average size of 1.1±0.6 kb from *Apotrechus* acinar labial glands. Analysis of over 100 clones identified 63 putative cDNAs, 22 of which had significant homology to sequences present in GenBank's non-redundant DNA database (E<0.05), mostly to proteins with house-keeping functions (see table S2). A single exception was the sequence encoding an amylase that was detected in fluid-filled reservoirs. Of the other 41 cDNAs that did not have significant homology to known sequences, 17 encoded one of four silk proteins (see below).

**Figure 4:**
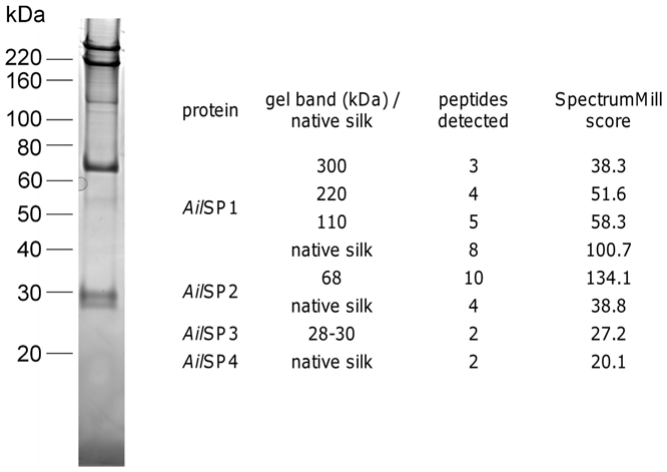
Identification of silk proteins by LC/MS. Silk proteins separated by SDS-PAGE of solubilized silk-webs are shown on the left. The number of peptides detected and the SpectrumMill score for each band are shown on the right. Spectrum Mill scores higher than 20 indicate a confident identification.

Solubilized silk-webs contained major protein bands at approximately 300, 220, and 68 kDa and fainter bands at 120, 30 and 28 kDa (figure 4). Similar protein bands were obtained from silk produced by different individuals and regardless of whether or not protease inhibitors were included during solubilization or reducing agents added during SDS-PAGE. Individual protein bands from SDS-PAGE gels or solid silk-webs were digested with proteases and the resulting peptides were analysed using mass spectroscopy. Comparison of experimentally-derived peptide masses with the predicted masses of *in silico* digested sequences encoded by the labial gland cDNA library showed that the bands at 300, 220 and 120 kDa all corresponded to a single protein sequence, partially encoded by three cDNA clones (figure 4). We named this protein *Ail*SP1 (*Apotrechus illawarra* Silk Protein 1; GenBank accessions JF508439 and JF508440). The longest *Ail*SP1 cDNA contained 1282 nucleotides of coding region, a stop codon, a 3′ untranslated region of 315 nucleotides, and a poly-A tail. Two of the *Ail*SP1 cDNAs differed by only by a single silent polymorphism (GenBank accession JF508439) while the third contained 77 single nucleotide polymorphisms (31 silent) and a trinucleotide insert (GenBank accession JF508440). The partial sequence of *Ail*SP1 consisted of an internal repetitive region of 37 or 38 residue repeats followed by a 163 residue C-terminal region that contained a single cysteine residue (figure S2). The small amino acids alanine, glycine and serine made up 24.3%, 23.4%, and 18.5% of residues respectively.

Three other silk proteins that occur at lower abundance were identified. The 68 kDa protein corresponded to a protein sequence we named *Ail*SP2 (figure 4; GenBank accession JF508441). *Ail*SP2 was encoded by 10 cDNA clones, seven of which began with a predicted signal sequence (SignalP 3.0 HMM p = 0.999) and ended with a stop codon, 3′ untranslated region, and poly-A tail, and were presumed to encode the full-length protein sequence. The predicted mature sequence of *Ail*SP2 comprised 404 amino acids and had a predicted mass of 41.4 kDa. The 28 and 30 kDa protein bands both corresponded to a protein sequence we named *Ail*SP3 (figure 4; GenBank accession JF508442), represented by 3 cDNA clones. The longest of these clones encoded a predicted signal sequence (SignalP3.0 HMM P = 0.982), stop codon, 3′ untranslated region and poly-A tail. The predicted mature *Ail*SP3 protein had 203 residues (14 of them cysteine) and a predicted mass of 22.4 kDa. A final silk protein, *Ail*SP4 (GenBank accession JF508443) was identified in silk-webs but not in the solubilized silk (figure 4). *Ail*SP4 was represented by a single partial 1.8 kb cDNA sequence that consisted of a partial coding region, putative stop codon, 3′ untranslated region, and poly-A tail. The partial protein sequence of *Ail*SP4 is high in proline (11.2%) and serine (19.9%) compared to glycine (9.9%) and alanine (7.8%).

## Discussion

The raspy cricket species used in this study produced shelters by joining leaves together with silk-webs. Given that the silk-webs were not air-tight, they are unlikely to be effective in preventing desiccation or the ingress of parasites in the wild, and the most likely function is to reduce predation. Silk-webs were found to be made of protein, and visual observations of crickets fabricating silk suggested that the labial glands might function as silk glands. Our identification of transcripts encoding silk proteins in labial glands confirms this directly.

The silk gland consists of acini connected by a network of ducts to the insect's salivarium. Silk glands from species that make cocoons have large lumens that store silk proteins in preparation for a short period of intense silk production [27]. In contrast, raspy cricket gland lumens are small, the amount of silk required for shelters is low, and animals probably produce silk as required. A reservoir attached to the common duct immediately before it joins the salivarium does not contain silk proteins but does contain amylase, suggesting that it has a salivary function.

**Figure 5:**
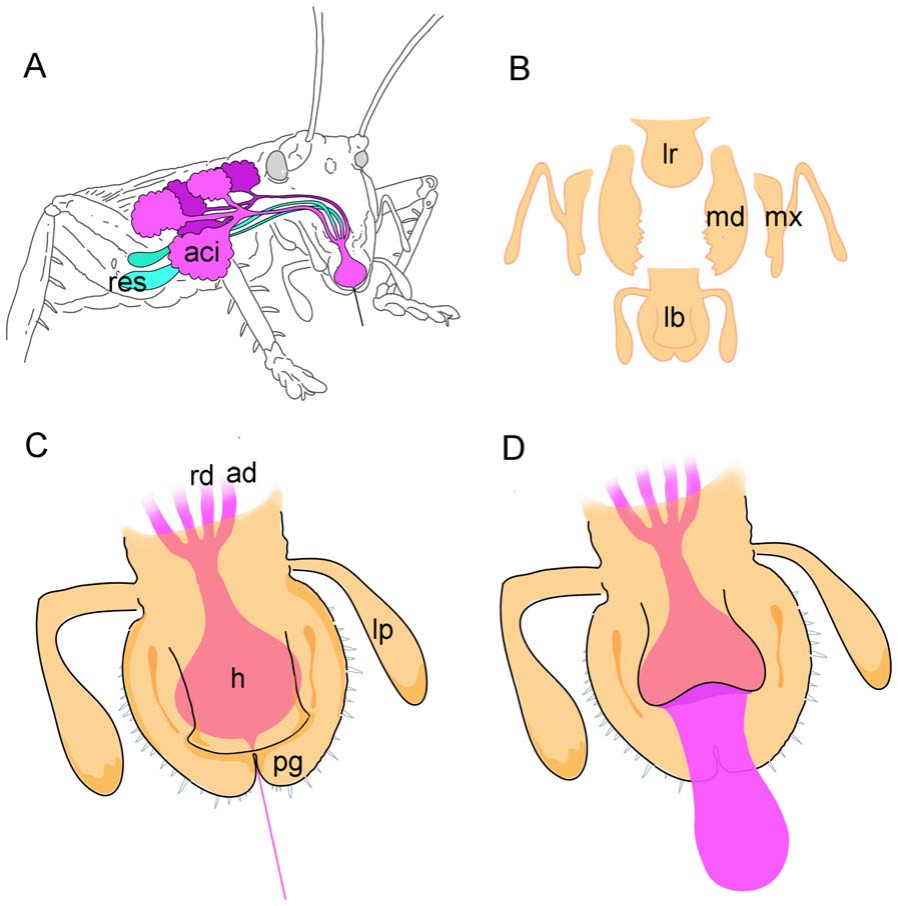
Fabrication of fibers and films by raspy crickets. (*a*) Indicative position of labial gland acini (aci) and reservoirs (res) and associated ducts. (*b*) Breakdown of raspy cricket mouthparts, including labium (lb), maxillae (mx), mandibles (md) and labrum (lr). (*c*) Expanded view of the labium with the hypopharynx (h) in closed position, forming a draw-down taper with the labial paraglossae (pg) to allow fiber fabrication. Liquid silk in the salivarium chamber between paraglossae and hypopharynx is shown in purple. lp = labial palp; rd = reservoir duct; ad = acinar duct. (d) Expanded view of the labium with the hypopharynx in open position, allowing silk dope to flow over paraglossae, allowing fabrication of films.

The silk-webs consisted of fibers and films, with the fibers providing the mechanical backbone of the webs and the films serving to glue the fibers to other building materials and to each other. Micro-Raman spectra indicated that the proteins present in fibers and films are indistinguishable at the levels of amino acid composition and secondary protein structure, suggesting they are made from the same protein solution. We propose that the anatomical arrangement in raspy crickets is specialised to be able to produce fibers and films interchangeably. Instead of having an external, tubular spinneret like lepidopterans and spiders [28], [29] the labial ducts of raspy crickets end in an aperture that is too large to act as a draw-down taper. Instead, silk dope probably exists in a liquid state in the salivarium chamber created by tucking the hypopharynx under the raised margins of the labial paraglossae. Single fibers are formed by drawing through the taper at the extremity of the labium, between the tips of the two paraglossae and the hypopharynx (figure 5). Opening the labium and hypopharynx by muscular control allows the insect to deposit silk dope in globules, which dry into films. Both films and fibers are found in the silks of other insects, including sawflies, honeybees, hornets and some beetles, e.g. [30].

The major silk protein *Ail*SP1 has evolved convergent features to those of the dominant fibrous proteins from silkworm cocoon and spider dragline silks: it is large, contains a high proportion of small amino acids, and adopts primarily an extended beta-sheet conformation in the mature silk. The spacing between beta-sheets in the side group direction is 1.27 nm, within the range reported for lepidopteran beta-sheet silks (0.93–1.57 nm) and slightly greater than that of polyalanine (1.06 nm) [31], [32]. If crystallites are formed from the repeat units of *Ail*SP1 this is a reasonable estimate, since 27% of residues in repeat regions are glycine, 27% are alanine, and 18% are serine, with the remainder larger residues. The presence of a cysteine residue in the C-terminus of *Ail*SP1 and the high frequency of cysteines in *Ail*SP3, as well as the observation that reducing agents are required for silk solubilization, suggest that disulfide bonding plays a structurally important role in cricket silk.

If raspy crickets produce silk films by depositing globules of liquid silk dope which are subsequently allowed to dry, then proteins in films are exposed to minimal shear and compression forces. Since the secondary structure of the proteins in fibres and films is essentially the same, shear forces cannot be a primary mechanism driving beta-sheet formation, as it is for the proteins in silkworm cocoon fibers [33]. Some insect species produce silk consisting of proteins with alpha-helical [34] and cross-beta [35] molecular structures, which shear forces are likely to disrupt rather than favour. In these species, the final molecular structure must form due to some combination of dehydration and the ordering of proteins within the gland prior to silk fabrication. Similar mechanisms may drive beta-sheet formation by raspy cricket silk proteins. Although not required for beta-sheet formation, shear force most likely accounts for the long range alignment of proteins within the fibers demonstrated by WAXS patterns and by birefringence measurements.

A comparison between raspy cricket silk and two unrelated insect silks, produced by silkworms and webspinners, is shown in Table 2. The three types of insects have been faced with a similar problem, the need to produce an insoluble and stable building material, and have independently evolved solutions with some convergent features. All three insect families produce silk proteins, with primary fibroin sizes ranging from moderately large to enormous, and effective size further increased due to cysteine cross-linking. The fibroins are rich in particular amino acids and in repetitive motifs that foster formation of extensive intermolecular beta-sheets, which assemble into insoluble and stable beta-sheet crystallites. On the other hand, there is wide variation between the three silks in regard to the gland of production, form of spinneret, and morphology of the mature silk product. Convergent evolution of silks has occurred at the molecular level; however the silk fabrication process and its anatomical substrates are highly flexible and idiosyncratic.

**Table 2:** Comparison of silks composed of beta-sheet forming proteins.

	Silkworm	Webspinner	Raspy cricket
Major silk fibroin size (kDa)	390 [39]	∼67 [40]	220, 300
Fibroin composition	46% Gly, 30% Ala, 12% Ser [39]	44% Gly, 31% Ser [41]	19% Ala, 19% Gly, 17% Ser
Repetitive sequence motifs	Yes [39]	Yes [42]	Yes
Dominant protein structure	beta-sheet [42]	beta-sheet [41]	beta-sheet
Role for cysteine cross-links	Yes [43]	Yes [40]	Yes
Silk gland	Labial gland [17]	Dermal glands on tarsi [44]	Labial gland
Spinneret	Rigid tube on labium [45]	Tubular bristles [44]	Movable labium-hypopharynx
Form of mature silk	Double fiber	Multiple fine fibers [41]	Fiber or film

## Methods and Methods

### Insects

Raspy crickets of the species *Apotrechus illawarra* and *Hyalogryllacris species 9* were collected from Meroo National Park, NSW, Australia. Crickets were housed in plastic jars with water and orthopteran food mixture [36]. They were supplied either with natural building materials (dry leaves, sticks and sand) or black plastic card. Non-gryllacridid crickets used for comparison were field crickets, *Acheta domestica* (Pisces Enterprises, Kenmore, Queensland, Australia), katydids of the genus *Conocephalus* (collected at the Meroo National Park field site) and white-kneed king crickets, *Penalva flavocalceata* (Minibeast Wildlife, Brisbane, Australia). Species used for comparison were dissected immediately following collection or receipt.

### Microscopy

Silk glands and mouthparts from adult insects of either sex were dissected and fixed in phosphate buffered saline (PBS) pH 7.0 containing 2% gluteraldehyde (Invitrogen, Carlsbad, USA) and examined using light microscopy. Silk-webs were mounted onto a stub using conductive tape, sputter-coated with gold and visualised under high vacuum on a Zeiss Evo LS15 scanning electron microscopy (SEM; Zeiss, Jena, Germany). Silk glands from a single large *Apotrechus* nymph were examined on a confocal scanning laser microscope (Carl Zeiss LSM5, Microimaging GmbH, Jena, Germany). The tissues were dissected and fixed in 4% formaldehyde in PBS for 10 minutes, washed three times for 30 minutes each in PBS, permeabilized for 1 hour in PBT (0.25% BSA, 0.4% Triton X in PBS), blocked for 1 hr in PBT-NGS (2% normal goat serum in PBT). After fixation, tissues were stained overnight at 4°C in 6.6 µM Alexa Fluor 568 phalloidin (Invitrogen, Carlsbad, USA), 1 µg/ml diamidino-2-phenylindole (DAPI; Invitrogen, Carlsbad, USA) in PBS. Before mounting, stained tissue was washed four times for 10 minutes each in PBS and placed in 70% glycerol, 2% propyl gallate in PBS for one hour in the dark.

Fiber birefringence was quantified using a Leica M205C polarising light microscope (Leica, Wetzlar, Germany) with a full wave compensator installed underneath the sample stage, so that its slow axis was aligned at +45° between the crossed polarising filters. Fiber diameters were measured using the Leica LAS software and birefringence calculated with reference to a Leica colour chart.

### Raman Spectroscopy

Raman spectra were obtained using an inVia confocal microscope system (Renishaw, Gloucestershire, UK) with 514 nm excitation from an argon ion laser through a ×50 (0.75 na) objective. Incident laser power was 4.5 mW and coaxial backscatter geometry was employed. Spectra were collected over the range 3200 to 100 cm^−1^ and averaged over at least 5 scans, each with an accumulation time of 20 seconds. Raman shifts were calibrated using the 520 cm^−1^ line of a silicon wafer. The spectral resolution was ∼1 cm^−1^. Fibers were analysed aligned parallel to the direction of laser polarisation with the use of a rotating stage. The final spectra used for analysis were averages of spectra collected from 6–10 different areas. All spectra were normalized on the CH_2_ and CH_3_ deformation modes at 1452 cm^−1^ that are attributable to amino acid side chains and thus not sensitive to protein conformation.

### X-ray scattering

Parallel bundles of *Apotrechus* silk fibers were analysed on the SAXS/WAXS beamline of the Australian Synchrotron (Melbourne, Australia). A wavelength of 0.124 nm and a nominal sample to detector distance of 0.559 m provided a *q*-range (*q* = 4πsinθ/λ) of approximately 0.01 to 0.22 nm^−1^, which was calibrated using a silver behenate standard. Samples were mounted in air, perpendicular to the beam, with scattering patterns collected in transmission. An optical microscope alignment system was used to accurately position samples in the X-ray beam. A background profile obtained without a sample was subtracted from experimental profiles to account for air scattering. Peak positions were measured using the Australian Synchrotron's 15ID SAXS/WAXS software.

### cDNA library construction

A cDNA library was constructed from the labial glands of four large *Apotrechus illawarra* nymphs. To induce silk production, the shelters of these crickets were removed and they were supplied with fresh building materials. One day later the insects were dissected in PBS pH 7.0 and the acinar parts of the labial glands removed and stored in RNAlater (Ambion, Austin, USA). Total RNA was prepared using RNAqueous-4PCR (Ambion, Austin, USA) and mRNA isolated using Micro-FastTrack 2.0 (Invitrogen, Carlsbad, USA). The cDNA library was created using Cloneminer II cDNA Library Construction kit (Invitrogen, Carlsbad, USA) according to the manufacturer's instructions. Clones containing cDNA inserts larger than 1 kb were sequenced by Micromon Services (Monash University, Melbourne, Australia) using an Applied Biosystems 3730S Genetic Analyser and Applied Biosystems PRISM BigDye Terminator Mix cycling chemistry. A library of possible protein sequences was generated *in silico* using EMBOSS Transeq [37]. Putative cDNAs were identified by the presence of a poly-A tail longer than 15 continuous nucleotides and/or the presence of an open reading frame longer than 300 bp. Signal peptides were predicted using the SignalP 3.0 algorithm [38].

### Mass spectroscopy

Cricket silk was solubilized in saturated lithium bromide with 5% 2-mercaptoethanol (both from Sigma-Aldrich, St. Louis, USA) at 95°C for one hour, after which no solid material could be observed. Silk-webs were not soluble in guanidinium hydrochloride or sodium dodecyl sulphate (SDS) supplemented with 2-mercaptoethanol, or in saturated lithium bromide in the absence of a reducing agent. In some experiments the denaturing solution included a protease inhibitor cocktail (Complete mini EDTA-free; Roche Applied Science, Penzberg, Germany). Lithium bromide was removed from solubilized samples by repeated concentration and dilution using a Centricon-10 centrifugal filter device (Millipore, Belerica, USA). Solubilized cricket silk proteins were separated by polyacrylamide gel electrophoresis (PAGE) using NuPage 4–12% Bis-Tris gels and 2-(N-morpholino)ethanesulphonic acid running buffer (Invitrogen, Carlsbad, USA) and stained with Coomassie Brilliant Blue (Sigma-Aldrich, St. Louis, USA). Protein gel bands or solid silk samples were digested with either trypsin or α-chymotrypsin (Sigma-Aldrich, St. Louis, USA) and analysed by reversed phase liquid chromatography coupled by electrospray ionisation to ion trap tandem mass spectrometry as previously described [34]. Mass spectral data sets were analysed using Spectrum Mill software (Agilent, Santa Clara, USA).

## Supporting Information

Figure S1 **WAXS pattern from** ***Apotrechus*** **silk fiber bundle.** Fiber axis is vertical. The d-spacings of scattering peaks are marked.
(TIF)

Figure S2 **Raspy cricket silk protein sequences.** Conserved repeats in *Ail*SP1 are highlighted in yellow, and predicted signal peptides for *Ail*SP2 and *Ail*SP3 are highlighted in green. Because disulfide bonding has a structural role in cricket silk, cysteine residues are highlighted in blue.
(TIF)

Video S1 ***Hyalogryllacris species 9*** **fabricating silk.** This video appears at 6× actual speed.
(WMV)

Table S1 **Proteins identified in fluid-filled reservoirs by LC/MS.**
(DOCX)

Table S2 **Assignment of** ***Apotrechus illawarra*** **cDNA non-silk library sequences.**
(DOCX)
