# Supplementary figures and images for: Silk from Crickets: A New Twist on Spinning
Source: PLoS One. 2012 Feb 15;7(2):e30408. doi: 10.1371/journal.pone.0030408 (PMC3280245; doi:10.1371/journal.pone.0030408)

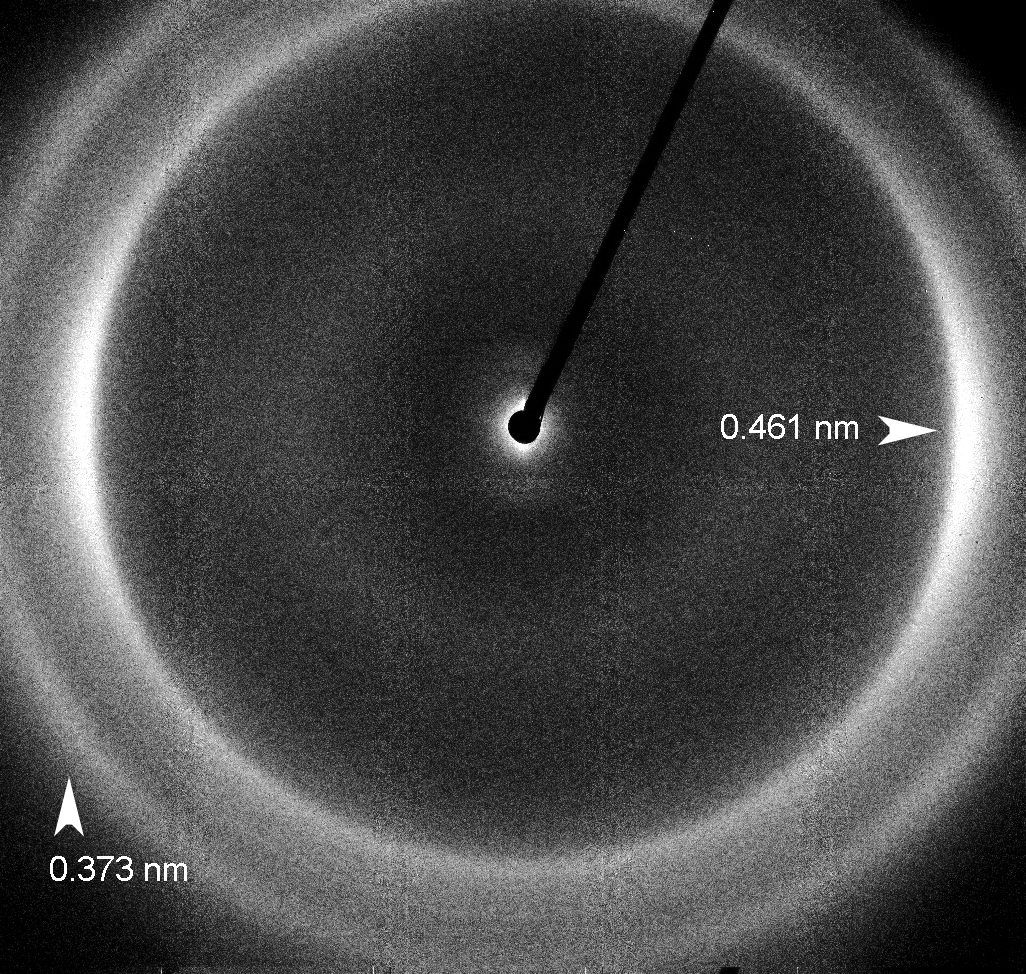

Supplement: Figure S1 — WAXS pattern from Apotrechus silk fiber bundle. Fiber axis is vertical. The d-spacings of scattering peaks are marked. (TIF) [file pone.0030408.s001.tif]

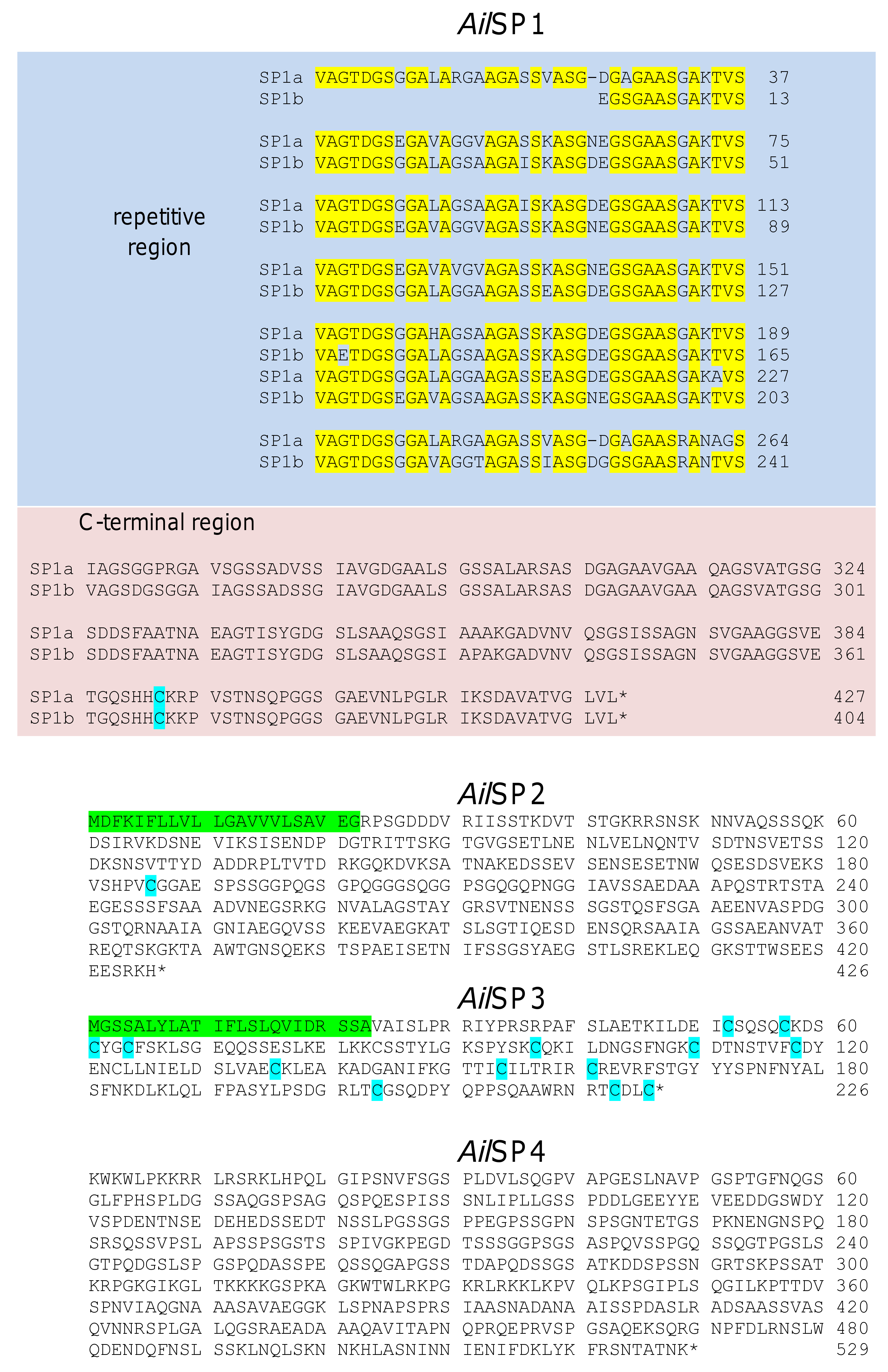

Supplement: Figure S2 — Raspy cricket silk protein sequences. Conserved repeats in AilSP1 are highlighted in yellow, and predicted signal peptides for AilSP2 and AilSP3 are highlighted in green. Because disulfide bonding has a structural role in cricket silk, cysteine residues are highlighted in blue. (TIF) [file pone.0030408.s002.tif]
