# Supplementary material for: Silk from Crickets: A New Twist on Spinning
Source: PLoS One. 2012 Feb 15;7(2):e30408. doi: 10.1371/journal.pone.0030408 (PMC3280245; doi:10.1371/journal.pone.0030408)
Supplement: Table S1 — Proteins identified in fluid-filled reservoirs by LC/MS. (DOCX) [file pone.0030408.s004.docx]

**Table S1. Proteins identified in fluid-filled reservoirs by LC/MS.**

| protein match | # of peptides | SpectrumMill score |
| --- | --- | --- |
| GA_JG443736 (alpha-amylase homology)^1^ | 3 | 37.6 |
|  |  |  |
| tubulin alpha chain^2^ | 5 | 63.4 |
| tubulin beta chain^2^ | 2 | 40.9 |
| actin 42A^2^ | 5 | 68.2 |
| actin A2^2^ | 2 | 23.8 |

^1^from *Apotrechus in silico* translated cDNA library

^2^from Genbank’s non-redundant protein database
