# Supplementary material for: Silk from Crickets: A New Twist on Spinning
Source: PLoS One. 2012 Feb 15;7(2):e30408. doi: 10.1371/journal.pone.0030408 (PMC3280245; doi:10.1371/journal.pone.0030408)
Supplement: Table S2 — Assignment of Apotrechus illawarra cDNA non-silk library sequences. (DOCX) [file pone.0030408.s005.docx]

**Table S2. Assignment of *Apotrechus illawarra* cDNA non-silk library sequences.**

| Genbank accession | description |
| --- | --- |
| JG443731 | Putative cDNA encoding protein similar to glucose dehydrogenase |
| JG443732 | Putative cDNA encoding protein similar to proteasome subunit |
| JG443733 | Putative cDNA |
| JG443734 | Putative cDNA |
| JG443735 | Putative cDNA encoding protein similar to calreticulin |
| JG443736 | Putative cDNA encoding protein similar to alpha-amylase |
| JG443737 | Putative cDNA encoding protein similar to gluteraldehyde-3-phosphate dehydrogenase |
| JG443738 | Putative cDNA |
| JG443739 | Putative cDNA |
| JG443740 | Putative cDNA |
| JG443741 | Putative cDNA encoding protein similar to glucose dehydrogenase |
| JG443742 | Putative cDNA encoding protein similar to glucose dehydrogenase |
| JG443743 | Putative cDNA |
| JG443744 | Putative cDNA |
| JG443745 | Putative cDNA |
| JG443746 | Putative cDNA |
| JG443747 | Putative cDNA encoding protein similar to Rab/RAS |
| JG443748 | Putative cDNA |
| JG443749 | Putative cDNA encoding protein similar to glucose dehydrogenase |
| JG443750 | Putative cDNA |
| JG443751 | Putative cDNA encoding protein similar to V-type ATPase |
| JG443752 | Putative cDNA encoding protein similar to elongation factor 1 alpha |
| JG443753 | Putative cDNA encoding protein similar to lipase |
| JG443754 | Putative cDNA |
| JG443755 | Putative cDNA encoding protein similar to B-cell associated protein (Bap) |
| JG443756 | Putative cDNA |
| JG443757 | Putative cDNA |
| JG443758 | Putative cDNA |
| JG443759 | Putative cDNA encoding protein similar to beta-tubulin |
| JG443760 | Putative cDNA |
| JG443761 | Putative cDNA |
| JG443762 | Putative cDNA encoding protein similar to glucose dehydrogenase |
| JG443763 | Putative cDNA |
| JG443764 | Putative cDNA |
| JG443765 | Putative cDNA |
| JG443766 | Putative cDNA |
| JG443767 | Putative cDNA |
| JG443768 | Putative cDNA encoding protein similar to elongation factor 1 alpha |
| JG443769 | Putative cDNA encoding protein similar to elongation factor 2 |
| JG443770 | Putative cDNA encoding protein similar to ribosomal protein s27A |
| JG443771 | Putative cDNA encoding protein similar to Na^+^/K^+^ ATPase |
| JG443772 | Putative cDNA encoding protein similar to methyltransferase |
| JG443773 | Putative cDNA encoding protein similar to myosin or zipper |
| JG443774 | Putative cDNA |
| JG443775 | Putative cDNA |
| JG443776 | Putative cDNA |
| JG443777 | Putative cDNA |
| JG443778 | Putative cDNA encoding protein similar to ribosomal protein L7 |
| JG443779 | Putative cDNA |
| JG443781 | Putative cDNA |
| JG443782 | Putative cDNA |
